# Identifying the Ion Channels Responsible for Signaling Gastro-Intestinal Based Pain

**DOI:** 10.3390/ph3092768

**Published:** 2010-08-26

**Authors:** Stuart M. Brierley, Patrick A. Hughes, Andrea M. Harrington, Grigori Y. Rychkov, L. Ashley Blackshaw

**Affiliations:** 1 Nerve-Gut Research Laboratory, Department of Gastroenterology & Hepatology, Hanson Institute, Royal Adelaide Hospital, Adelaide, South Australia, Australia 5000; 2 Disciplines of Medicine, The University of Adelaide, Adelaide, South Australia, Australia 5000; E-Mail: patrick.hughes@adelaide.edu.au (P.A.H.); andrea.harrington@adelaide.edu.au (A.M.H.); ashley.blackshaw@adelaide.edu.au (L.A.B.); 3 Disciplines of Physiology, The University of Adelaide, Adelaide, South Australia, Australia 5000; E-Mail: grigori.rychkov@adelaide.edu.au (G.Y.R.)

**Keywords:** TRPA1, TRPV4, TRPV1, ASICs, Na_V_ and K_V_ channels, visceral pain

## Abstract

We are normally unaware of the complex signalling events which continuously occur within our internal organs. Most of us only become cognisant when sensations of hunger, fullness, urgency or gas arise. However, for patients with organic and functional bowel disorders pain is an unpleasant and often debilitating reminder. Furthermore, chronic pain still represents a large unmet need for clinical treatment. Consequently, chronic pain has a considerable economic impact on health care systems and the afflicted individuals. In order to address this need we must understand how symptoms are generated within the gut, the molecular pathways responsible for generating these signals and how this process changes in disease states.

## 1. Gastrointestinal Pain

Pain and discomfort originating from the gastrointestinal tract are common and often debilitating complaints of patients with functional and organic gastrointestinal disorders. Functional gastrointestinal disorders are highly prevalent, affecting up to 30% of the population. These disorders lack an underlying organic disease that readily explains their symptoms and encapsulate patients with Non Cardiac Chest Pain [1,2], Functional Dyspepsia [3,4,5,6] and Irritable Bowel Syndrome [7,8,9,10]. Organic disorders such as Inflammatory Bowel Disease (which includes Crohn’s Disease and Ulcerative Colitis) are chronic relapsing and remitting inflammatory disorders of the gastrointestinal tract affecting ~3.6 million people in the USA and Europe alone [11,12,13,14]. Although the aetiology of these diseases are very different, visceral pain is the unifying link and more often than not the most poorly managed symptom. 

In the periphery activation of sensory nerve endings, which feed into nociceptive pathways of the central nervous system (CNS), give rise to the sensation of pain. The threshold for pain has to be high enough not to interfere with normal physiology but low enough that it can be evoked before marked tissue damage occurs [15]. In order to achieve this function “nociceptive” nerve endings express a variety of ion channels and receptors which transduce mechanical and chemical stimuli or regulate neuronal excitability [16,17,18,19,20,21]. Acute “physiological” pain arising from the gastrointestinal tract normally serves as an alarm system that is activated in response to excessive distension or intense muscular contractions to warn of impending damage. Unlike acute pain, inflammatory pain is caused by injury, irritation or infection and acts as a warning to prevent further damage. Frequent, re-occurring “chronic” pain often manifests from an underlying disease [22] and a failure of nociceptors to “reset”. Typically, reduced sensory thresholds and increased afferent responsiveness to mechanical stimuli underlie pain signalling in a variety of gut based inflammatory and chronic pain syndromes [23]. It is becoming clear that the mechanisms underlying acute, inflammatory and chronic pain are varied but originate from changes at the level of the periphery [24,25,26,27,28]. Despite a growing list of ion channels responsible for signalling acute “physiological” pain we are only beginning to determine the channels responsible for inflammatory and chronic visceral pain in animal models and translating these findings to human diseases. This article will review very recent advances in this field which have brought specific ion channels into the spotlight for pain originating from the gut.

## 2. Sensory Afferents Innervating the Gastrointestinal Tract

Visceral sensory nerves follow three main anatomical pathways from the gastrointestinal tract to the CNS - the vagal, splanchnic and pelvic nerves [20]. Vagal afferent fibres have neuronal cell bodies in the nodose and jugular ganglia. Splanchnic and pelvic fibres have cell bodies in either the spinal thoracolumbar and lumbosacral dorsal root ganglia (DRG) [17,20,29]. Correspondingly, vagal and spinal afferent neurons project centrally to either the brainstem or spinal cord respectively. The peripheral projections of these neurons terminate at various levels within the gut wall, including the mucosa, enteric ganglia, muscle layers, serosa and mesenteric attachments [17,29]. 

**Figure 1:**
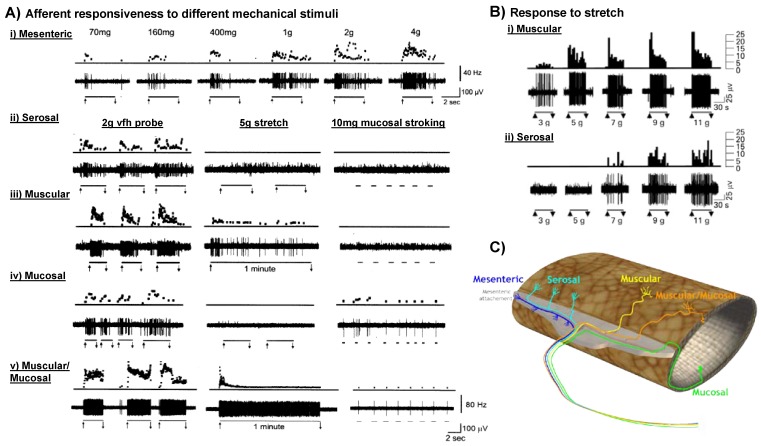
Five classes of mechanosensitive afferent innervate the murine gut. (**A**). Afferents are classified by their responsiveness to distinct mechanical stimuli, circular stretch, fine mucosal stroking and focal compression with calibrated von Frey hairs (vfh): (**A i**) Mesenteric and (**A ii**) serosal afferents respond in a graded manner to focal compression with probes and high intensity stretch (≥7 g; see (**B ii**)) or distension (>35 mmHg; not shown). They do not respond to low intensity mucosal stroking (10 mg; see A (**B ii**)) or low intensity circular stretch (≤5 g; also see (**B ii**)); (**B iii**). Muscular afferents (or tension sensitive afferents as they are called in the upper gut) respond to low intensity circular stretch (<3 g; also see (**B i**)) but not fine mucosal stroking; (**B iv**). Mucosal afferents have the lowest mechanical activation thresholds of all afferent classes, responding to fine mucosal stroking (10 mg von Frey hair), but do not respond to circular stretch; (**B v**). Muscular/mucosal afferents display the properties of muscular and mucosal afferents as they respond to both low intensity stretch and fine mucosal stroking. Overall, muscular, mucosal and muscular/mucosal afferents have low-thresholds for mechanical activation via stretch or stroking respectively, whilst serosal and mesenteric afferents have high-thresholds for mechanical activation by stretch; (**C**). Graphical representation of the 5 different subtypes of afferent innervating the mouse colorectum. (**A**) Modified from Brierley *et al.* [31] with permission; (**B**) Modified from Hughes *et al.* [37] with permission; (**C**) Modified from Brierley [18] with permission.

A wide assortment of afferents have been recorded and described from throughout the entire length of the gut, with numerous experimental techniques and mechanical stimuli used to classify their function. Historically fibres have been classified by a low- or high-threshold of activation to organ distension [30], however, not all fibres respond to distension or can be adequately classified based on this stimulus alone [31,32,33,34]. In the mouse gastrointestinal tract the picture is somewhat clearer, with afferent endings classified into five subtypes according to their functional responsiveness to select stimuli and the proposed location of their mechanoreceptive fields [31,34] (Figure 1). These classes are termed mucosal, muscular (or tension receptor in the upper gut), muscular/mucosal, serosal and mesenteric afferents (Figure 1) [31,34,35,36,37]. Mucosal afferents respond to fine tactile stimulation of the luminal (mucosal) surface and anatomically appear as bare endings in the lamina propria of villi [38]. Muscular afferents (or tension receptors) respond to distension at physiological levels (<20 mmHg or low intensity stretch stimuli <3 g; Figure 1) [34,37] and consist of specialized terminals which surround myenteric ganglia termed Intraganglionic Laminar Endings (IGLEs) [39,40,41,42]. The responsiveness of vagal tension receptors quickly saturates at around ~20 mmHg, whilst spinal muscular afferents respond linearly over wide-distension pressures (10–60 mmHg) [36,37,43,44]. In the colon muscular afferents are prevalent in the pelvic pathway but rare in the splanchnic pathway [31,40]. Muscular/mucosal afferents are exclusive to the pelvic pathway and respond to both tactile and distension stimuli (Figure 1), but their anatomy is yet to be demonstrated [31]. Serosal and mesenteric afferents respond at noxious levels of distension (>35 mmHg) [31,45,46] or higher intensity circular stretch (>7 g; Figure 1) [36,37] and in other species correspond with varicose branching axons which surround blood vessels [47]. A separate mechanically-insensitive class of fibre has also been described which respond only to chemical stimuli [48,49]. 

## 3. What Is a Gut-Innervating Nociceptor?

This may seem an obvious question, but gut afferent nomenclature and function remain contentious issues. In part, this is due to the historical lack of a standardised nomenclature system for defining subtypes. Unlike cutaneous afferents, which are classed on nerve fibre conduction velocity and distinct response properties [50], the majority of gut afferents have conduction velocities in the C-fibre range (<1 m/s) [35,36,51,52,53] and therefore cannot be segregated on this parameter alone. As such defining gut afferent subtypes based on mechanical responsiveness to select stimuli (see above) has allowed progress in this area. Despite these recent classifications there is still great debate as to which afferent subtypes signal “nociceptive” events within the gut.

Vagal afferents are generally associated with signaling physiological events rather than painful events, although there are exceptions notably in the oesophagus [54], whereas spinal pathways are generally associated with sensations of pain, bloating, discomfort and urgency [20]. Of the spinal afferents the most obvious nociceptive suspects are the high-threshold (serosal and mesenteric) afferents as they are activated at distension pressures (threshold ~35 mmHg) [36,37] that are reportedly at the start of the noxious range [55]. However, it has also been suggested that low-threshold (muscular and muscular/mucosal) afferents can also signal pain, as although they signal physiological events they can also encode into the nociceptive range [30,55]. 

Despite the apparent controversy surrounding the identity of gut innervating nociceptors, one might suggest that effective therapies for visceral pain should preferentially target the higher-threshold subtypes of afferent. This would be potentially advantageous, allowing specific targeting of events signalled at noxious levels. Although low-threshold afferents can encode into the noxious range one would suspect that targeting these afferents would also affect their responsiveness to lower intensity events and therefore adversely affect normal perception and reflex function.

## 4. Visceral Hypersensitivity

Visceral hypersensitivity is apparent in numerous Functional Gastrointestinal Disorders [23]. As such, investigators have used varying visceral pain models to try and mimic these diseases in an effort to determine which afferent subtypes are altered [56]. Most of the controversy centres on the colonic pathways and although there is consensus that inflammation does change afferent function there are discrepancies in the specific details. These studies show absent or inconsistent effects of inflammation on low-threshold distension-sensitive afferents [57,58,59]. However, in the mouse colon and rectum the picture is somewhat clearer with distinct afferent subtypes and pathways involved over different time-courses [21,37]. 

**Figure 2:**
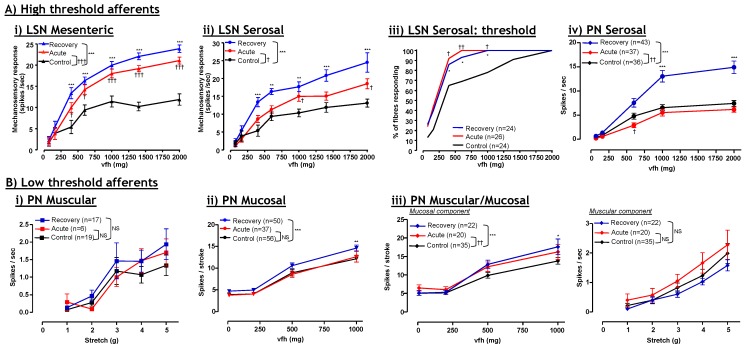
Specific classes of murine mechanosensitive colonic afferent become hypersensitive during and after inflammation. TNBS-induced colitis causes pronounced mechanical hypersensitivity in (**A**) high-threshold splanchnic (LSN): (**A i**) mesenteric afferents and (**A ii**) serosal afferents and (**A iii**) increases the percentage of afferents responding at a given stimulus intensity. These effects on mechanosensitivity become more apparent after resolution of inflammation (recovery; 30 days post-TNBS administration) when compared with acute conditions (7 days post-TNBS); (**A iv**) By contrast, pelvic (PN) high-threshold serosal afferents only become hypersensitivity following resolution from inflammation (recovery); (**B**) Low-threshold afferents display little or no change in mechanosensitivity after TNBS-induced colitis. Minor, but significant increases in mucosal responsiveness are observed in (**B ii**) mucosal and (**B iii**) muscular/mucosal afferents. By contrast, (**B i**) muscular and (**B iii**) muscular/mucosal afferent responses to stretch are unaffected by inflammation in the mouse. Modified from Hughes *et al.*, [37] with permission.

Inflammatory hypersensitivity of splanchnic afferents with high mechanosensory thresholds (serosal and mesenteric) but not those with low-thresholds (mucosal and muscular) (Figure 2). Furthermore, the extent of this hypersensitivity is greater after recovery from overt tissue inflammation [21,37]. Pelvic afferents are not hypersensitive during acute inflammation, however, pelvic serosal and mucosal afferents become hypersensitive following recovery from inflammation [21,37]. Overall, these observations give further credence to the suggestion that effective therapies for visceral pain should preferentially target the higher-threshold subtypes of afferent, as these afferents display the greatest mechanical hypersensitivity during and after inflammation. It is now becoming apparent that certain ion channels have differential expression amongst these afferent subtypes, which underlies their overall function and their contribution to pathophysiology.

## 5. TRPV1

In peripheral afferent terminals TRPV1 acts as an immediate sensory alarm in response to low pH, heat and noxious agents such as capsaicin [60,61,62]. As such TRPV1 is probably the best know member of the TRP channel family, is the archetypal somatic pain sensor and one of the most heavily studied channels in the gut. However, compared with cutaneous afferents the role of TRPV1 in the viscera is not straightforward as its expression is not necessarily indicative of sensory neuron "nociceptor" function. Firstly, TRPV1 is also expressed in non-neuronal gut tissues, such as the oesophageal mucosa [63]. Secondly, all classes of low and high-threshold afferents in vagal and spinal pathways respond, in varying degrees, to capsaicin [33,53,64,65,66]. In particular, there is a high functional expression of TRPV1 in all colonic afferent subtypes [52,53,65,66]. In fact TRPV1 expression is greater in colonic innervating neurons than those innervating the skin with as many as 80% of colonic splanchnic colonic neurons expressing TRPV1 [52,53,65,67,68]. Thirdly, acute capsaicin application can subsequently decrease [49], increase [53] or have no effect on mechanosensitivity [49,64], depending on the class of gut afferent studied. 

**Figure 3:**
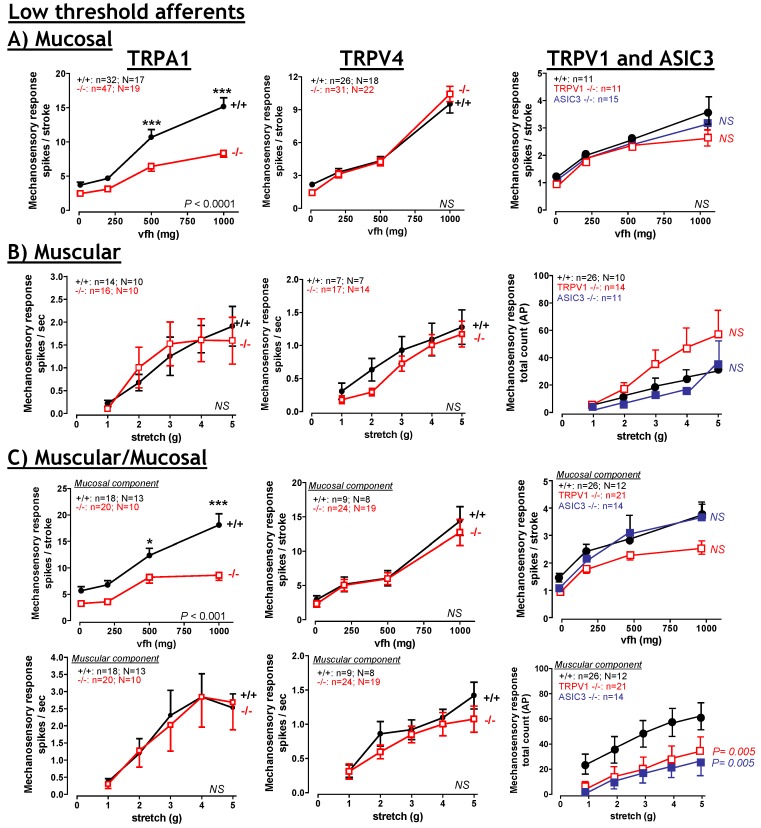
Limited contribution of TRPA1, TRPV4, TRPV1 and ASIC3 to low-threshold afferent mechanosensitivity. TRPA1 deletion only reduces the responsiveness of (**A**) mucosal and (**C**) muscular/mucosal afferents to mucosal stroking and does not affect the stretch responses of (**B**) muscular or (**C**) muscular/mucosal afferents. Deletion of TRPV4 has no effect at all on low threshold afferent mechanosensitivity (**A–C**). Deletion of TRPV1 or ASIC3 markedly reduces the stretch response of (**C**) muscular/mucosal afferents. However,TRPV1 or ASIC3 deletion has no effect on mucosal or muscular afferent mechanosensitivity (**A**, **B**). TRPV4 modified from Brierley *et al.*, [36] with permission. TRPA1 modified from Brierley *et al.*, [35] with permission. TRPV1 and ASIC3 modified from Jones *et al.*, [52] with permission.

Stark contrasts between somatic and visceral afferents are also apparent when investigating mechanosensory function in TRPV1^–/–^ mice. Deletion of TRPV1 has no effect on somatic mechanosensory function [69]; however deficits are apparent in intestinal afferents, specifically certain types of low-threshold distension sensitive gastro-oesophageal, jejunal and pelvic colonic afferents (Figure 3) [51,52,70]. By contrast, high-threshold gut afferents are unaffected by TRPV1 deletion [52,70]. Nevertheless, TRPV1 has been strongly implicated in contributing to visceral pain. The ability of TRPV1 to respond to low pH [61] places it in a prime position to sense oesophageal acid reflux, giving rise to the sensation of heartburn, whilst intra-oesophageal capsaicin instillation induces symptoms of epigastric burning [71]. In animal models intra-colonic capsaicin administration causes pronounced visceral pain [72,73], whilst TRPV1^–/–^ mice also display decreased visceromotor responses (VMR) to colorectal balloon distension (CRD) [52], suggesting an involvement of TRPV1 in signalling behavioural aspects of colonic pain. 

Numerous inflammatory mediators can interact with TRP channels to modulate their functional properties (see Table 1) [74]. TRPV1 is no exception and in colonic innervating DRG neurons 5-HT can sensitize TRPV1 function [75]. Specifically, 5-HT incubation increases capsaicin- and proton-evoked currents by 1.6- and 4.7-fold respectively. 5-HT also decreases the activation threshold temperature of TRPV1 from 42 to 38°C. Interestingly, these effects can be mimicked by 5-HT_2_ and 5-HT_4 _receptor agonists. Correspondingly, this sensitisation of TRPV1 can be reduced by selective 5-HT_2_ and 5-HT_4_ receptor antagonists but not by a 5-HT3 receptor antagonist [75]. Activation of another class of receptor, Protease Activated Receptor-2 (PAR_2_) can also sensitize TRPV1, increasing capsaicin currents over time following activation of PAR_2_ with a Protease Activated Receptor_2_-Activating Peptide (PAR_2_-AP) [76,77]. PARs also interact with other TRP channels as discussed below.

**Table 1:** Summarizes the ion channels implicated in signaling gut based “physiological” pain in healthy conditions and details where (gut innervating neurons, gut innervating afferents or whole animal gut based recordings) this function was demonstrated. This table also documents interactions of these channels with inflammatory mediators, specifically those identified within gut neurons or gut afferents. √: Indicates a demonstrated role for this channel in gut neurons, gut afferents or whole animal gut based recordings; X: Indicates no demonstrated role for this channel; *ND*: indicates the function of this channel has not been determined in the setting specified; ↑ indicates the inflammatory mediator increases channel function; ↓ indicates the inflammatory mediator decreases channel function; ↔ indicates the inflammatory mediator has no effect on channel function.

Functional role for channel in healthy:	Isolated gut neurons	Afferent gut fibres	Whole animal	Is channel function in gut neurons altered by inflammatory mediators?
TRPV1	√	√	√	√; 5-HT ↑, PAR_2 _↑
TRPV4	√	√	√	√; 5-HT ↑, PAR_2 _↑, Histamine ↑, PAR_4_↓
TRPA1	√	√	√	√; Bradykinin ↑, 4-HNE ↑, PAR_2_↔ or ↑
ASIC1	*ND*	√	√	*ND*
ASIC2	*ND*	√	√	*ND*
ASIC3	*ND*	√	√	*ND*
Na_V_1.5	X	ND	ND	*ND*
Na_V_1.7	√	ND	ND	*ND*
Na_V_1.8	√	ND	√	√; TNFα ↑
Na_V_1.9	√	ND	X	ND
Kv	√	ND	ND	√; TNFα ↓

### 5.1. TRPV1: Contribution to Inflammation

A perhaps somewhat overlooked function of TRPV1 activation is its ability to cause neurogenic inflammation, via local release of substance P and CGRP. Mice lacking TRPV1 develop significantly less oesophagitis after acid exposure compared with TRPV1^+/+^ mice [78], whilst administration of a TRPV1 antagonist before or after intracolonic TNBS administration significantly reduces colitis severity [79,80]. Similarly, in rats and mice TRPV1 antagonists attenuate disease severity in dextran sulphate sodium (DSS)-induced colitis [81,82]. However, diametrically opposed to these observations there are also reports of TRPV1 having a protective role in inflammatory models, with TRPV1 activation improving DSS-induced colitis in mice [83]. Furthermore, a dinitrobenzene sulfonic acid model causes more severe colitis in TRPV1^–/–^ mice than TRPV1^+/+^ mice [84].

### 5.2. TRPV1: Contribution to Visceral Hyperalgesia

In addition to contributing to inflammation TRPV1 also mediates the development and short term maintenance of colonic hypersensitivity in non-inflammatory and neonatal models [85,86,87]. However, the role of TRPV1 in colonic sensory afferents after the initial insult remains somewhat unclear and appears to depend on the model and indeed the exact time frame studied. In adult inflammatory models there appears to be a transient role for TRPV1, with initial increases in TRPV1 expression and function during the height of active colonic inflammation [57,80,88,89], which return to normal at later time points [80]. Paradoxically, visceral mechanical hypersensitivity still remains at this and longer time points [21,37,90,91]. Furthermore, TRPV1 deletion or the use of TRPV1 antagonists only partially reverses mechanical hypersensitivity and hyperalgesia [80,86] (Figure 5), indicating the involvement of other channels in colonic afferent hypersensitivity. Clearly some controversy exists and resolution is clouded due to species differences, the different hypersensitivity models used and also the time points at which studies take place. Identifying a clear contribution for TRPV1 in afferent hypersensitivity is further complicated by its ability to act as a polymodal sensor and its contribution to the inflammatory process. 

**Figure 4:**
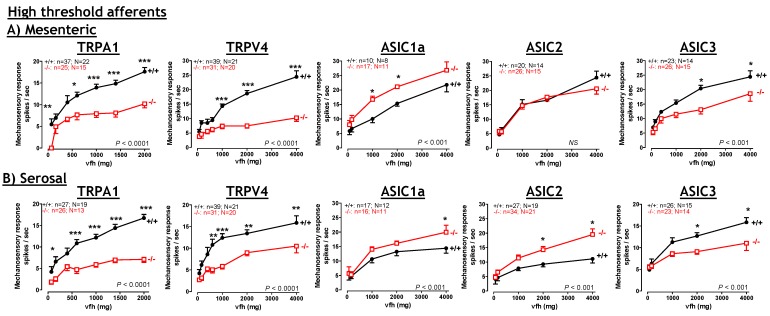
Significant contribution of TRPA1 and TRPV4 to normal colonic high-threshold afferent mechanosensory function. Deletion of TRPA1 or TRPV4 markedly reduces the mechanosensitivity of high-threshold colonic splanchnic (**A**) mesenteric and (**B**) serosal afferents. Deletion of ASIC3 also reduces afferent mechanosensitivity, but the effects are more modest (**A**,** B**). In stark contrast, ASIC1a and ASIC2 deletion either increases mechanosensitivity or causes no effect (**A**,** B**), indicating at best a modulatory role for these channels. Overall, by far the greatest deficits are apparent in the TRPV4^–/–^ and TRPA1^–/–^ afferents. TRPV4 modified from Brierley *et al.*, [36] with permission. TRPA1 modified from Brierley *et al.* [35] with permission. ASIC1a, ASIC2, ASIC3 modified from Page *et al.* [115] with permission.

### 5.3. TRPV1: Therapeutic potential?

Perhaps the best indicator for TRPV1s involvement in gut based pain comes from human patient studies which indicate increased expression or function of TRPV1 in Gastro-Oesoghageal Reflux Disease, Functional Dyspepsia, Inflammatory Bowel Disease and Irritable Bowel Syndrome (Table 2). The number of TRPV1 expressing fibres are increased in biopsies from patients with erosive reflux [92] and non-erosive reflux disease [93], whilst TRPV1 gene expression is increased in the oesophageal mucosa of both patient groups [94]. Functional Dyspepsia patients are hypersensitive to a capsaicin challenge [95,96], whilst a TRPV1 G315C polymorphism increases the susceptibility of developing Functional Dyspepsia [97]. Further down the gut, TRPV1 immunoreactive colonic nerve fibres are increased in patients with active Inflammatory Bowel Disease [98]. Studies of biopsies from patients with Irritable Bowel Syndrome show that mucosal fibres containing TRPV1 are increased and that this increase may correlate with symptoms [99]. Furthermore, patients with idiopathic rectal hypersensitivity have increased TRPV1 fibres in all layers of the gut wall [100].

**Table 2:** Summarizes the ion channels implicated in signaling gut based “inflammatory” or “chronic” pain and details where (gut innervating neurons, gut innervating afferents or whole animal gut based recordings) this function was demonstrated. This table also documents which channels have altered expression in gut based human pain syndromes. √: Indicates a demonstrated role for this channel in gut neurons, gut afferents or whole animal gut based recordings; X: Indicates no demonstrated role for this channel; *ND*: indicates the function of this channel has not been determined in the setting specified.

Altered channel function or expression in gut based inflammatory models?	Isolated gut neurons	Afferent gut fibres	Whole animal	Is channel expression or function altered in gut based human diseases?
TRPV1	√	√	√	√
TRPV4	√	√	√	√
TRPA1	*ND*	√	√	*ND*
ASIC1	*ND*	*ND*	*ND*	*ND*
ASIC2	*ND*	*ND*	*ND*	*ND*
ASIC3	*ND*	√	√	√
Na_V_1.5	*ND*	*ND*	*ND*	√
Na_V_1.7	X	*ND*	*ND*	√
Na_V_1.8	√	*ND*	√	*ND*
Na_V_1.9	X	*ND*	√	*ND*
Kv	√	*ND*	*ND*	*ND*

Although TRPV1 may appear the perfect target for pharmacotherapy these findings should be taken with a caveat. Most of these studies are conducted using human mucosal biopsies and the fibres innervating the mucosa are the least likely, at least in normal conditions, to signal pain due to their very low activation thresholds to mechanical stimuli. In addition to increased TRPV1 expression some studies have also reported that the density of neural innervation is also increased in patient biopsies. In particular significant increases in total nerve fibre counts, as indicated by PGP9.5 and substance P-immunoreactive fibres [99], is suggestive of axonal sprouting. As elegantly noted previously by Holzer the benefit of TRPV1 therapy may pivot around whether “TRPV1 up-regulation in gut disorders reflects a causal implication rather than just a marker of enhanced sensitivity and functionality of afferent neurons or enhanced arborisation of their fibres in the periphery” [101]. If the former is true, then it will be a tricky path to follow, as many different types of gut afferent express TRPV1. Therefore blocking TRPV1 may help with pain relief; however, it may also concurrently perturb normal physiology. These factors combined with the well documented complications of hyperthermic responses in clinical trials involving TRPV1 antagonists [102] raise a cautionary tale. Nevertheless, a recent study suggests targeting specific modalities of TRPV1 activation may overcome the hyperthermic response in animal models [103]. Additionally, TRPV1s involvement in neurogenic inflammation may suggest a “window of opportunity” whereby a TRPV1 antagonist may be beneficial in reducing the initial inflammatory response and preventing subsequent inflammation-induced changes in ion channel expression within gut sensory afferent neurons. Another possibility would be to target specific TRPV1 splice variants. For example, in the rat oesophageal epithelium there is a report of a TRPV1 splice variant which is significantly different (60 kDa *vs.* 95 kDa) to the sensory DRG form of TRPV1 [63]. Depending on the functionality of the 60 kDa protein, one could suggest it as a specific therapeutic target for the "first step" in acid detection in the oesophageal mucosa and therefore the subsequent acid reflux associated pain without the potential hyperthermic side-effects. Although this may treat the pain associated with reflux it would not address the underlying cause [104,105,106].

## 6. TRPA1

TRPA1 has been implicated as a mechanosor [107] and has recently emerged as a major mediator of inflammatory pain [108]. In gut innervating sensory neurons TRPA1 expression is more restricted than that observed for TRPV1. In these neurons TRPA1 is localised in both vagal and spinal afferents with 55% of vagal gastro-oesophageal afferent neurons, 54% of splanchnic colonic and 58% of pelvic colonic innervating DRG neurons expressing TRPA1 [35,109]. In peripheral tissue, TRPA1 is located in mucosal, serosal and mesenteric nerve fibres [35] indicating TRPA1 is well placed to participate in the function of these afferent subtypes. Correspondingly, TRPA1 agonists (mustard oil or cinnamaldehyde) universally evoke mechanical sensitisation of serosal and mesenteric [35] high-threshold [53] colonic afferents and pelvic mucosal afferents [35]. These compounds also activate colonic innervating DRG neurons in isolation [109]. The use of TRPA1^–/–^ mice has allowed specific determination of the afferent fibres types which utilize TRPA1 to detect mechanical stimuli. These studies show striking deficits occur in high-threshold colonic afferents, specifically splanchnic mesenteric afferents and splanchnic and pelvic serosal afferents (Figure 4) [35]. Furthermore, TRPA1 makes a modest contribution to low-threshold mucosal afferent mechanosensitivity in both vagal and pelvic pathways innervating the oesophagus/stomach and colon respectively. In contrast TRPA1 does not contribute to the mechanosensitivity of vagal tension receptors or pelvic muscular and muscular/mucosal afferents (Figure 3). Interestingly, these are the classes of afferent which are affected by TRPV1 deletion [51,52]. Another important finding is the observation that TRPA1 deletion increases the activation thresholds of serosal and mesenteric afferents to mechanical stimuli [35], suggesting TRPA1 is important in setting mechanical activation thresholds. The predominant mechanosensory role for TRPA1 in high-threshold serosal and mesenteric afferents combined with the ability of TRPA1 agonists to increase the mechanical responsiveness of these afferents is suggestive of an involvement in pain. Supporting this assertion TRPA1^–/–^ mice also display decreased VMR to high intensity (80 mmHg) CRD [35], but not at lower distension pressures (15–60 mmHg) [109]. The observation that intra-colonic administration of TRPA1 agonists enhances VMR to the higher distending pressures (45 and 60 mmHg) within 2 hours of administration [109] suggests TRPA1 can modulate visceral mechanical hyperalgesia (discussed below). Further inference can also be deduced by the observation that the classes of TRPA1^–/–^ afferent displaying deficits are exactly the same classes of TRPA1^+/+^ afferent displaying mechanical hypersensitivity after TNBS induced colitis, namely splanchnic mesenteric and serosal afferents and pelvic serosal and mucosal afferents (compare Figure 2, Figure 3 and Figure 4) [21,35,37]. 

### 6.1. TRPA1: Contribution to Inflammation

Mustard oil has long been used as a visceral inflammatory model to provoke tissue damage and sensitize nociceptors [73,110]. We now know that mustard oil (allyl-isothiocynate) is a TRPA1 agonist [111] and recent findings provide us with a more complete understanding of how this compound causes these effects in the viscera. TRPA1 agonists induce neurogenic inflammation via the release of substance P and CGRP [112] with increases in cytokines associated with macrophage and neutrophil activation and recruitment [113]. However, the contribution of TRPA1 to different types of inflammation may not be as prevalent as that of TRPV1, with recent reports suggesting the severity of colitis induced by TNBS is unaffected by TRPA1 deletion [109].

### 6.2. TRPA1: Contribution to visceral hyperalgesia.

TRPA1 is also directly activated by irritants such as the endogenous aldehyde, 4-Hydroxynonenal (4-HNE) [74,108,112]. Intracolonic administration of mustard oil or 4-HNE increases the VMR to CRD at the highest distending pressures (45 and 60 mmHg, respectively) within 2–3 hours. Correspondingly, these agonists also cause neuronal activation, as indicated by induced c-fos expression, in the superficial laminae (I and II) of thoracolumbar (splanchnic) and lumbosacral (pelvic) spinal cord in TRPA1^+/+^ but not TRPA1^–/–^ mice [109]. Thus intra-colonic administration of TRPA1 agonists activates spinal nociceptors and causes visceral hyperalgesia. TRPA1 is also indirectly activated by inflammatory mediators such as bradykinin and PARs [74,108,112]. TRPA1 mediates bradykinin-induced mechanical hypersensitivity in the guinea-pig oesophagus [114] and the bradykinin-induced mechanical hypersensitivity observed in splanchnic serosal afferents [35,48]. TRPA1 is also required for PAR_2_-induced visceral hyperalgesia [109] as the delayed visceral mechanical hypersensitivity to CRD caused by intracolonic administration of the PAR_2_-AP in TRPA1^+/+^ mice is completely absent in TRPA1^–/–^ mice. Although minor controversy exists because in splanchnic colonic serosal afferents the association between PAR_2_ and TRPV4 seems to predominate over PAR_2_/TRPA1 interactions [35] and may be related to the promiscuity of PAR_2_ interactions on numerous TRP channels (discussed below).

**Figure 5:**
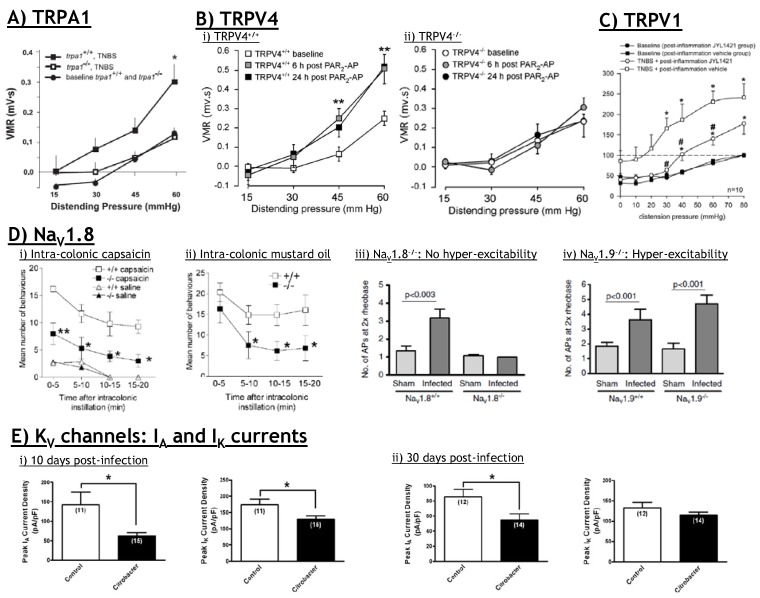
Multiple ion channels contribute to short and long term visceral hyperalgesia. (**A**) TRPA1 deletion reduces the mechanical hyperalgesia induced by TNBS colitis; (**B**) Mechanical hyperalgesia evoked by intra-colonic PAR_2_-AP administration in (**B i**) TRPV4^+/+^ mice is lost in ii) TRPV4^–/–^ mice; (**C**) Administration of a TRPV1 antagonist (JYL1421) after the induction of colitis with TNBS reduces mechanical hyperalgesia; (**D**) Deletion of Na_V_1.8 reduces both (**D i**) capsaicin and (**D ii**) mustard oil induced colonic hyperalgesia; (**D iii**) Na_V_1.8^–/–^ DRG neurons innervating the jejunum do not display the hyper-excitability induced by *Nippostrongylus brasiliensis* infection; (**D iv**) By contrast, Na_V_1.9^–/–^ neurons still display *Nippostrongylus brasiliensis* induced hyper-excitability; (**E**) K_V_ I_A_ and I_K_ currents are reduced in colonic innervating DRG neurons (**E i**) 10 days post-*Citrobacter rodentium* infection, whilst (**E ii**) K_V_ I_A_ currents are also reduced 30 days post-infection. (**A**) modified from Cattaruzza *et al.* [109], used with permission from The American Physiological Society; (**B**) modified from Sipe *et al.* [124] used with permission from The American Physiological Society; (**C**) modified from Miranda *et al.* [80] with permission; (**D**) i-ii) modified from Laird *et al.* [73] with permission. (**D iii-iv**) modified from Hillsley *et al.* [132] with permission; (**E**) modified from Ibeakanma *et al.* [133] with permission.

In addition to short term sensitisation, TRPA1 also has a consistent and longer term role in visceral hyperalgesia. TRPA1 agonists cause greater mechanical hypersensitivity in afferents from animals with acute TNBS-induced colitis compared with untreated mice, indicating an enhanced role for TRPA1 during inflammation [35]. In the whole animal, at 3 days post-TNBS administration, visceral hypersensitivity and increased neuronal c-fos expression in the spinal cord normally observed in TRPA1^+/+^ mice are completely absent in TRPA1^–/–^ mice (Figure 5) [109]. Furthermore, intrathecal injection of an antisense oligodeoxynucleotide, to down regulate TRPA1 expression, suppresses the colitis-induced hyperalgesia to nociceptive CRD and intracolonic mustard oil in rats studied 8 days post-TNBS [89].

### 6.3. TRPA1: Therapeutic potential?

Since intense focus to determine the role of TRPA1 has occurred only relatively recently there is a lack of studies to indicate whether or not its expression is altered in organic and functional bowel disorders. However, given the results documented from animal models it appears an extremely good candidate to target for the treatment of visceral pain. Although there is a mild contribution of TRPA1 to low-threshold afferents, its major contribution appears to occur in the high-threshold classes of afferent, their sensitisation by various mediators and the development of hyperalgesia. The contribution of TRPA1 to mucosal afferent mechanosensitivity may be beneficial in diarrhoea disorders such as diarrhoeal predominant IBS and Inflammatory Bowel Disease, since mucosal afferents are likely to play a role in initiating propulsive motility. There is also emerging evidence for involvement of TRPA1 in mediator release from enterochromaffin and enteroendocrine cells [116,117,118] and this function needs to be considered. Although a role for TRPA1 in hearing mechanotransduction was a major focus, this is less likely based on recent data [119,120] and potential therapeutic opportunities in pain may still be problematic due to a modulatory role for TRPA1 in most classes of somatic afferents [121,122]. Most recently a gain-of-function mutation in TRPA1 has been shown to cause Familial Episodic Pain Syndrome [123].

## 7. TRPV4

In gut innervating sensory DRG neurons TRPV4 expression is even more restricted than that observed for TRPA1. TRPV4 is localised in 38% of gastro-oesophageal vagal neurons [36], 65–76% of splanchnic colonic DRG neurons [36,124] and 58% of pelvic colonic DRG neurons [36]. Although TRPV4 expression is more restricted it is actually enriched within colonic DRG neurons, with its expression 20-fold greater in splanchnic colonic DRG neurons than in whole DRG. In the periphery TRPV4 protein co-localizes with CGRP in colonic nerve fibres in the serosal and mesenteric attachment. By contrast, TRPV4 expression is scarce in intramuscular or mucosal nerve fibres [36].

Consistent with the lack of TRPV4 expression in gastro-oesophageal vagal neurons deletion of TRPV4 has absolutely no effect on vagal afferent function [36]. However, in colonic afferents, where TRPV4 is enriched, mechanosensory responses are dramatically reduced in TRPV4^–/–^ mice. This is the case for both splanchnic serosal and mesenteric afferents (Figure 4). TRPV4 deletion also increases the mechanosensory thresholds of these afferents suggesting TRPV4, like TRPA1, contributes to setting mechanical activation thresholds. Furthermore, pelvic serosal afferents display similar deficits in mechanosensory responses and thresholds to those seen in the splanchnic pathway. As was the case in the vagal pathway low-threshold pelvic afferents are completely unaffected by TRPV4 deletion (Figure 3). Taken together this data indicates TRPV4 makes a specific and major contribution to high-threshold colonic afferent mechanosensory function. The extent of these deficits in mechanosensitivity are some of the largest reported in any afferent population, somatic or visceral [36]. These changes in colonic neuron function also translate to decreased VMR to CRD in TRPV4^–/–^ mice or in mice with down-regulated TRPV4 expression (via siRNA intrathecal injection), the extent of which is particularly apparent at higher distension pressures [36,124,125].

### 7.1. TRPV4: Contribution to visceral hyperalgesia.

The endogenous TRPV4 agonist 5,6-EET (an arachidonic acid metabolite) significantly potentiates mechanosensory responses in TRPV4^+/+^ mice, which is lost in TRPV4^–/–^ mice. Another TRPV4 agonist, 4α-PDD causes significant TRPV4 mediated calcium influx in isolated colonic DRG neurons [125]. Intracolonic administration of 4α-PDD in TRPV4^+/+^ mice increases neuronal c-fos expression in the lumbosacral spinal cord and causes dose-dependent visceral hypersensitivity [125]. Therefore, TRPV4’s role is not only linked to visceral mechanosensation but also to visceral hypersensitivity [36,124,125]. 

TRPV4 can be sensitised by a series of inflammatory mediators both *in vivo* and *in vitro*. Pre-exposure of colonic DRG neurons to histamine or 5-HT, increased Ca^2+^ responses induced by 4αPDD via various second messenger pathways [126]. 5-HT or histamine also enhances TRPV4 expression at the plasma membrane. Down-regulation of TRPV4, via siRNA intrathecal injection significantly reduces the hypersensitivity induced by 5-HT or histamine [126]. Although PAR_2_ interacts with TRPV1 and TRPA1 (see above) there is evidence for a strong interaction with TRPV4, which underlies visceral hypersensitivity [124,125]. TRPV4 and PAR_2_ are anatomically and functionally co-expressed in a large percentage of colonic innervating neurons [124,125]. In isolated colonic DRG neurons the PAR_2_-activating peptide, PAR_2_-AP, sensitizes TRPV4 mediated currents, whilst the PAR_2_-AP also activates splanchnic serosal colonic afferent fibres by a TRPV4-dependent mechanism [124]. Intra-colonic administration of PAR_2_-AP also causes enhanced VMR to CRD at higher distending pressures (>40 mmHg) measured 6 hours [124,125] and 24 hours [124] after administration. This is not observed in TRPV4^–/–^ mice at either time point [124,125], suggesting TRPV4 mediates both the acute and delayed hyperalgesia induced by PAR_2_ activation [124] (Figure 5). Taken together this data suggests TRPV4 is a common mechanism to several known mediators of visceral hypersensitivity, including, histamine, 5-HT and PAR_2_. Conversely, a related protease activated receptor, PAR_4 _is actually responsible for suppressing the excitability of colonic DRG neurons [127] whilst intracolonic PAR_4_ agonist administration significantly reduces PAR_2_ and TRPV4 agonist-induced allodynia and hyperalgesia in response to CRD [128].

### 7.2. TRPV4: Therapeutic potential?

At face value TRPV4 appears to be an ideal target for the treatment of visceral pain. TRPV4 has a high specificity for high-threshold spinal colonic afferents with one of the largest deficits in mechanosensory function documented in null mutant mice. There appears to be little to no role in low-threshold spinal afferents or in vagal afferent fibres. TRPV4 is concentrated in the outer layers of the colon and is association with blood vessels, in particular during active inflammation of the colon associated with Crohn’s disease and Ulcerative Colitis [36]. Furthermore, serine proteases levels [129,130], 5-HT signalling [131] and histamine [24,25,26] are all known to be increased in IBS patients and TRPV4 interacts with these mediators. However, unlike TRPV1 and TRPA1 currently there are no antagonists available for TRPV4. In terms of non-gut actions, TRPV4 contributes to the detection of osmotic stimuli [134,135,136] and also to somatic inflammatory pain [137,138,139,140,141].

## 8. Acid Sensing Ion Channels

ASICs are members of the DEG/ENaC family of ion channels which contribute to the detection of pH and mechanical stimuli. ASIC1, 2, and 3 mRNA expression has been detected in vagal and spinal pathways but their abundance varies greatly between gut innervating afferents [142,143]. ASIC1 has a greater expression in gastro-oesophageal innervating nodose ganglion neurons compared with colonic innervating neurons, similar levels of ASIC2 are in both sets of neurons [142,143], whilst colonic neurons contain more ASIC3 than gastro-oesophageal neurons [142,143]. Indeed as little as 30% of colonic neurons express ASIC1, whilst 47% express ASIC2 and 73% express ASIC3 [142,143]. Correspondingly, studies utilizing ASIC^–/–^ mice have demonstrated widespread differences in the mechanosensory mechanisms between different pathways. Deletion of ASIC1a increases the mechanical sensitivity of splanchnic colonic and vagal gastro-oesophageal afferents (Figure 4) [115,144]. Disruption of ASIC2 has varied effects: increasing mechanosensitivity in gastro-oesophageal mucosal endings, decreasing gastro-oesophageal tension receptors, increasing colonic serosal endings, and causing no change in colonic mesenteric endings (Figure 4) [115]. In ASIC3^–/–^ mice, gastro-oesophageal tension afferents have markedly reduced mechanosensitivity, whilst mucosal afferents are unchanged. In the colon splanchnic serosal and mesenteric afferents display mechanosensory deficits (Figure 4) [115], whilst in the pelvic nerve muscular/mucosal afferents have significantly reduced mechanosensory responses (Figure 3) [52]. Low-threshold pelvic mucosal and muscular afferents remained unchanged (Figure 3) [52]. Although the deficits in mechanosensitivity observed in the respective ASIC^–/–^ mice are fairly modest (Figure 3 and Figure 4), particularly compared with those documented for TRPV4 and TRPA1, the effects on afferent mechanosensitivity translate to changes in the whole animal. Observations of altered gastric emptying and faecal output are found in ASIC1a^–/–^ and ASIC2^–/–^ mice respectively [115,144], whilst reductions in colonic mechanosensory function in ASIC3^–/–^ mice [52,115] translate to significantly reduced VMR to CRD [52]. Differences between the upper gut and lower gut afferents are also apparent in the sensitivity to the non-selective DEG/ENaC blocker benzamil. Splanchnic colonic afferent mechanosensitivity is virtually abolished by benzamil application, whereas gastro-oesophageal afferents are only marginally inhibited. Deletion of ASIC2 or ASIC3 significantly reduces the ability of benzamil to reduce splanchnic serosal afferent mechanosensitivity, whilst deletion of ASIC1a has no effect on benzamil sensitivity [143]. ASIC3 also contributes to visceral peripheral sensitization as demonstrated by an intra-colonic zymosan induced increase in VMR in control mice which is lost in ASIC3^–/–^ mice [86]. Deletion of ASIC3 also prevents gastritis-induced acid hyper-responsiveness of the stomach-brainstem axis [145].

### 8.1. ASICs: Therapeutic Potential?

ASICs form heteromultimers [146,147] and the composition of these heteromultimers differs depending on the tissue studied [18]. This is reflected in benzamil having a greater effect on colonic afferents than vagal afferents and the predominant expression of ASIC3 in splanchnic colonic neurons and ASIC1 transcripts in gastro-oesophageal afferents [143]. In the gut only ASIC3 makes a positive contribution to mechanosensitivity, a conclusion that is emphasized by its key role in colonic peripheral sensitization. In humans increased ASIC3 expression is observed in Crohn's disease intestine [148]. Therefore, targeting specific heteromultimers, those which heavily express ASIC3, could be beneficial, although the role of ASIC3 in vagal and somatic mechanoreceptors has to be taken into consideration. Potent ASIC3 blockers are becoming available [149,150] which is promising, however, they need to be extremely selective given that ASIC1a and ASIC2 are involved in CNS and baroreceptor function [151,152].

## 9. Voltage Gated Sodium and Potassium Channels (Na_V_ and K_V_)

These channels are critical in determining the excitability of sensory neurons, with Voltage Gated Sodium Channels (Na_V_) mediating the rapid upstroke of the action potential, whilst Voltage Gated Potassium Channels (K_V_) act to repolarise the cell membrane, limiting repetitive firing. Na_V_ channels have distinct electrophysiological and pharmacological properties. This review will focus on Na_V_1.7, Na_V_1.8 and Na_V_1.9, for detailed reviews see [16,153,154,155,156]. Na_V_1.7 mediates a Tetrodotoxin (TTX)-sensitive current important in setting action potential generation threshold. Na_V_1.8 is a TTX-resistant current contributing to the upstroke of action potentials and to continuous firing activity during prolonged depolarisations. Na_V_1.9 mediates a persistent current that contributes to setting resting membrane potential [155]. In the gut, colonic innervating thoracolumbar DRG neurons contain transcripts for Na_V_1.7, Na_V_1.8 and Na_V_1.9 [157] although functionally most exhibit Na_V_1.8 and NaV1.7 like currents with few exhibiting currents similar to those mediated by Na_V_1.9 [158]. Voltage-gated Potassium channels (K_V_) comprise various subunits [159] and physiologically are broadly classed on their inactivation kinetics as either delayed rectifier (I_K_) currents or transient outward (I_A_) currents [16]. 

### 9.1. Na_V_ and K_V_: Contribution to Visceral Hyperalgesia.

It is clear from most studies utilizing inflammatory, nematode or bacterial models that gut innervating neurons become hyper-excitable after the initial insult. This is apparent in neurons innervating the stomach [160,161,162,163], small intestine [132,164,165,166] and the colon [133,157,158]. As such these changes are consistent across different regions of gut and across different experimental models. This hyper-excitability is characterized by a decreased threshold for activation, increased firing rate and changes in Na_V_ and K_V _channels. Specifically, these changes relate to an increase in TTX-resistant Na_V_ channel function and suppression of I_A_ and I_K_ currents. Recent reports indicate a crucial role for Na_V_1.8 in colonic innervating DRG neurons and that its expression is differentially regulated across varying time points during colitis [157]. Furthermore, nematode induced jejunal neuronal hyperexcitability is lost in Na_V_1.8^–/–^ mice but not Na_V_1.9^–/–^ mice (Figure 5) [132]. Correspondingly, at the level of the whole animal, Na_V_1.8^–/–^ mice show blunted pain and hyperalgesia responses to intra-colonic capsaicin (TRPV1 agonist) or mustard oil (TRPA1 agonist) (Figure 5) [73]. Although Na_V_1.9^–/–^ mice display normal acute pain responses they display reduced inflammation-induced visceral hyperalgesia [167]. In terms of K_V_, I_A_ and I_K_ currents are reduced in colonic innervating DRG neurons 10 days post-*Citrobacter rodentium* infection, whilst suppression of K_V_ I_A_ currents contributes to neuronal hyper-excitability 30 days post-infection (Figure 5) [133].

**Figure 6:**
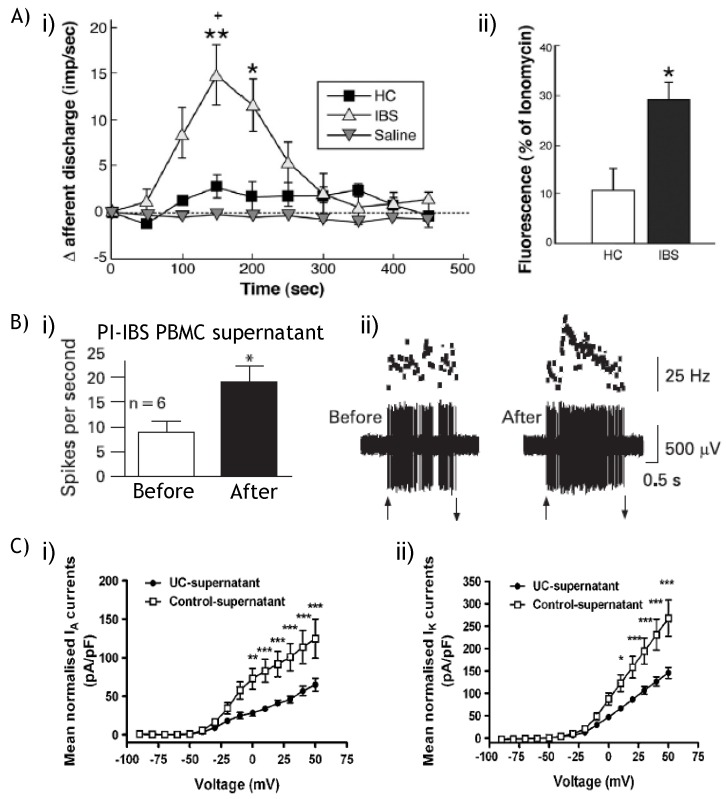
Supernatants from visceral pain patients alter visceral afferent function. (**A**) (**A i**) Mucosal supernatants obtained from colonic mucosal biopsies of IBS patients increase the nerve discharge of rat jejunal afferents compared with healthy controls (HC) or saline alone; (**A ii**) When tested on isolated DRG neurons the IBS supernatant-induced increases in intracellular Ca^2+^ were significantly higher than those obtained with supernatants from healthy control (HC) patients. Modified from Barbara *et al.* [26] with permission; (**B**) (**B i**) Peripheral blood mononuclear cell (PBMC) supernatants from post-infectious diarrhoea-predominant Irritable Bowel Syndrome (PI-IBS) patients cause a marked increase in the mechanosensory response of mouse pelvic colonic serosal afferents; (**B ii**) Original record on the mechanosensory response of a pelvic serosal afferent before and after PI-IBS supernatants. Modified from Hughes *et al.* [168] with permission; (**C**) Supernatants obtained from colonic biopsies from Ulcerative Colitis (UC) patients cause significant decreases in colonic DRG neuron (**C i**) K_V_ I_A_ currents and (**C ii**) I_K_ currents compared with control supernatant. Modified from Ibeakanma and Vanner [169] with permission.

Most recently it has been shown that the pro-inflammatory cytokine Tumour Necrosis Factor-α (TNFα) enhances Na_V_1.8 currents and suppresses I_A_ and I_K_ currents to evoke hyper-excitability in colonic DRG neurons (Figure 6). 

These TNFα induced effects on Na_V_ and K_V_ currents are similar to those observed during TNBS-induced colitis [169]. Colonic biopsies from Ulcerative Colitis patients contain increased levels of TNFα. Correspondingly, supernatants from these biopsies also caused neuronal hyperexcitability via enhancement of Na_V_1.8 currents and suppression of K_V_ I_A_ and I_K_ currents. Similarly, mucosal supernatants obtained from the colonic mucosal biopsies of IBS patients increase nerve discharge of rat jejunal afferents and cause greater supernatant-induced increases in intracellular Ca^2+^ compared with those from healthy control patients (Figure 6) [26]. Furthermore, it has also been demonstrated that peripheral blood mononuclear cells from post-infectious diarrhoea-predominant IBS patients also have elevated cytokine levels [27] and supernatants from these cells cause mechanical hypersensitivity in splanchnic and pelvic colonic afferents (Figure 6) [168].

### 9.2. Na_V_ and K_V_: Therapeutic potential?

Although Na_V_1.8 is an obvious choice as it appears to play a crucial role in visceral hyper-excitability in animal models, as yet there are no documented reports of altered function or expression in gut pain syndromes. However, other Na_V_s have been implicated for example IBS patients with mutations in *SCN5A* (the gene encoding Na_V_1.5) are more likely to report gastrointestinal symptoms, especially abdominal pain [170]. A Na_V_1.7 gain-of-function mutation in *SCN9A*, is linked to the human condition *“familial rectal pain syndrome”* now renamed *“paroxysmal extreme pain disorder”*. As the name suggests it is characterised by rectal and abdominal pain commonly associated with defecation [171,172,173]. However, there is a lack of data to indicate which afferents are actually associated with this condition and paradoxically the hypersensitivity in colonic DRG neurons in animal inflammatory models appears to involve Na_V_1.8 and not Na_V_1.7 [157,158]. Furthermore in another condition, idiopathic rectal hypersensitivity, colonic specimens display more nerve fibres expressing Na_V_1.7 [174]. Although intra-rectal lidocaine administration has been shown to reduce abdominal pain associated with diarrhoea-predominant IBS [175] knowing which Na_V_ to target in gut pain syndromes remains a difficult question. Therefore targeting mediators, such as TNFα, which interact with these channels may be a therapeutic option, particularly in Ulcerative Colitis patients [169] and post-infectious diarrhoea-predominant IBS patients [27,168]. The specific identity of the K_V_ subunits implicated in the inflammation induced suppression of I_A_ and I_K_ currents remains unreported.

In terms of somatic systems Na_V_1.8 is highly expressed in cutaneous nociceptors [176,177] and Na_V_1.7, Na_V_1.8 and Na_V_1.9 are all implicated in somatic pain, but again it depends on the model used. Neuropathic pain develops normally in mice lacking both Na_V_1.7 and Na_V_1.8 [178], however Na_V_1.7, Na_V_1.8 and Na_V_1.9 are critical in inflammation associated pain [179,180,181,182]. In another human disorder, loss-of-function *SCN9A* (Na_V_1.7) mutations cause a complete insensitivity to pain [183].

## 10. Concluding Remarks

Despite the apparent controversy concerning what constitutes a gut innervating nociceptor it is becoming clear from the literature that a variety of ion channels contribute to signalling visceral pain and hyperalgesia in a variety of conditions. Unique combinations of channels are expressed on high-threshold gut afferents and this may provide potential new pharmacological strategies for the management of pain and hyperalgesia originating from the gastrointestinal tract. To turn this potential into reality we must determine if promising targets from animal models translate into altered channel expression and function in human visceral pain disorders, whilst determining unequivocally that these changes are the underlying basis of visceral hyperalgesia. 
